# Textile Fiber Pollution: Relating Textile Features to Fiber Release in Pilling Experiments

**DOI:** 10.1021/acsomega.4c09501

**Published:** 2025-05-29

**Authors:** Mercedes Pereira, Jorge López-Beceiro, Ana-María Díaz-Díaz, Laura S. Vázquez, Ramón Artiaga

**Affiliations:** Centro de Investigacións en Tecnoloxías Navais e Industriais, 16737 Universidade da Coruña, Campus Industrial de Ferrol, Ferrol 15403, Spain

## Abstract

The concern regarding microplastic pollution has increased in recent years, including microfibers which come from textiles. It is well-known that wear and tear of clothes produce fiber loss, resulting in a loss of their aesthetic and physical requirements and contributing to environmental pollution. Some properties of fibers such as their nature and length have been related to fiber losing from clothes. The aim of this work is to evaluate the contribution of a few physical, dynamic, and thermo-mechanical properties of the textiles on the susceptibility to fiber release: dimensional features at the level of fiber, yarn, and fabric, and the fabric modulus and its strain under heat. Thermal and mechanical characterization is performed in a dynamic mechanical thermal analyzer. Fiber release along time is evaluated using a pilling machine. Experimental results are analyzed by principal component analysis.

## Graphical abstract

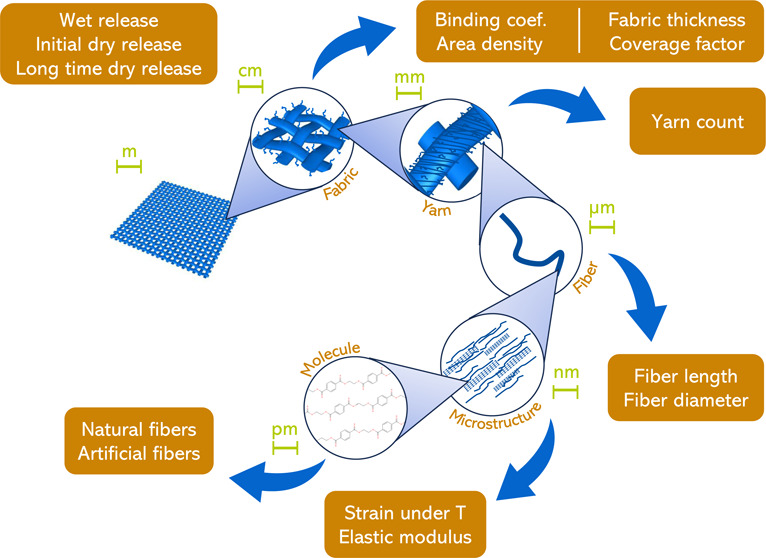

## 1 Introduction

The concern regarding microplastics has been a constant in recent years in environmental studies; however, it is a relatively new problematic. Even though plastic consumption has been growing since the 1940s, the wastes were directly discarded until the decade of 1980, and it was not until 10 years later that plastic started to be recycled.^1^ Figure 1 shows the number of results we obtained after searching in Google scholar for the topic “microplastics”, filtering by intervals of 5 years since 1950. It reveals that it was not until the current century that the number of hits per segment increased above 1000. Since the year 2000, the interest has grown exponentially, leading to about 20,000 hits in the last two years and a half.

**Figure 1:**
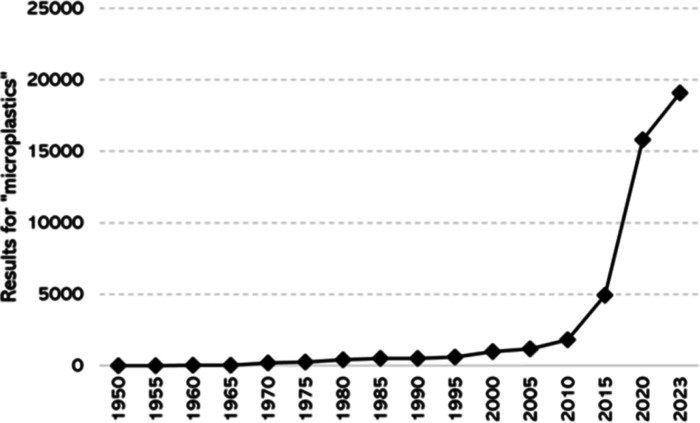
Results for “microplastics” in Google scholar for periods of 5 years since 1950 until 2023, consulted in June 2023.

Nowadays it is known that microplastics comprehend the vast majority of the plastic in the oceans and that they are mostly found in the shape of fibers. The role of textile industry in this problem is important as the 14% of the global waste generation is composed of these products, which are responsible for the release of around 0.2–0.5 million tonnes of microfibers to the seas.^2^

The emissions of textile microfibers have their origin in the industrial manufacture of the garments and other textiles, their daily use and, especially, their maintenance: mechanical drying and washing.^3^ Thus, the fiber release from the garments can be produced in two different environments: wet conditions and dry conditions. The former corresponds to the maintenance of textiles during washing and is more widely studied as it is generally considered to have a greater contribution to the release of microfibers. This arises from the fact that microfibers in dryers can be collected, while the filters of the conventional washing machines are not able to retain most of the released fibers still maintaining adequate conditions for the washing itself.^4^ Some studies have found that the amount of fibers released during drying is lower than that obtained in washing.^4,5^ However, the fiber release produced both in dryers and in normal daily use of the textiles also contributes to the contamination with microfibers and, collaterally, to increase the amount of textile waste as it reduces the service life of the garments. Indeed, it was recently proposed that this direct release to air has equal importance as the release in water,^6^ and Pirc et al. reported a higher release during drying than that in washing.^7^ Cummins et al. recently found some evidence that waterborne microfiber pollution has condenser tumble dryers as an important contributor, while vented dryers contribute also to airborne pollution.^8^ A deep understanding of the shedding behavior under these conditions and the features that can be improved could help to create textiles with a longer life which produce less contamination during its service life.

One of the strategies to reduce contamination with microfibers is the use of traps and filters in the drums of the washing machines. Commercial products have been developed to retain fibers during washing such as the GuppyFriend and Cora Ball studied by Kärkkäinen and Sillanpää.^4^ However, as they conclude, those solutions do not retain the main amount of the released fibers, which leads to a situation in which the textile fibers are able to be released to the environment, although in a slight less extent. In the case of tumble dryers, the filters included in the machines are able to retain more fibers. However, it has been demonstrated that microfibers end up in the environment as well due to the escape through the filter pores and also to the cleaning process of the filters.^9^

The understanding of a complex behavior such as the release of fibers in textiles has become a matter of concern since there are many variables implicated in the process. There are several studies that analyze the properties of the fabrics in fiber loss behavior, such as molecular nature and organization,^10^ the arrangement of fibers in different classes of yarns,^11^ or at the fabric structure level.^12^ Others compared the physical and chemical properties with the fiber release.^13^ Moreover, there are some recent studies such as the review made by Palacios-Marín and Tausif^14^ and the one of Rathinamoorthy and Raja Balasaraswathi^15^ that provide a clear overview of the results on this field. However, as they conclude, it is difficult to compare the fiber release of textiles produced under different conditions, and the variables concerning the different levels of structural arrangement of the textiles must be considered. In addition to those compositional and structure features, thermal and mechanical properties are important as they are derived from the nature and structure of these materials, for that they are susceptible to affect the shedding behavior.

Attending to this challenging situation, this study analyzes a group of properties of the textiles at the different levels of their structure (fiber, yarn, and fabric) together with the nature of the material and its dynamic and thermo-mechanical behavior. The textiles are subjected to fiber release experiments, and their properties are related to their shedding behavior using principal component analysis (PCA). This statistical method allows one to obtain an overview of the whole situation by reducing the dimensions of the implicated variables by projecting them in the main directions of variance.

## 2 Materials and Methods

### 2.1 Materials

The materials used in this study are 15 commercial woven fabrics made of different blends of polyester (PET) with acrylic, elastane (EL), cotton (CO), viscose (VI), and wool (WO). Regarding the weave pattern of these fabrics, 5 are taffeta, 7 are twill, and 3 are satin, as indicated in Table 1. All fabrics were kindly provided by the Inditex company.

**Table 1:** Composition and Dimensional Features of the Woven Fabrics To Be Tested

				fiber	yarn count^c^	fabric	
fabric	description (substrate)	composition	*fiber type other features*	length^a^ diameter^b^	warp weft	grammage^d^ thickness^a^	coverage factor binding coef.
ATMF	suedette (satin)	PET	continuous	∞	187	243	100
			*raised*	14.9	367	0.52	0.40
CRMF	crimp (taffeta)	PET	continuous	∞	78	50	51
			*texturized, twisted*	23.4	79	0.13	1
CRPMF	crepe (twill)	94% PET	continuous	∞	234	207	86.3
		6% EL	*twisted*	11.3	223	0.36	0.67
CYMF	corduroy (taffeta)	70% PET	continuous	∞	301	289	99.1
		25% EL	*texturized and intermingled*	14.4	212	1.3	1
		5% acrylic					
DNMF	denim (twill)	55% PET	staple	32–38	482	302	99.9
		40% CO	*intermingled with EL*	13.4	510	0.72	0.5
		5% EL					
DXMF	double weave (taffeta)	78% PET	staple	40–45	195	257	98.7
		28% VI	*double weave*	13.7	269	0.62	1
		4% EL					
JAWMF	jackward (satin)	60% PET	continuous	∞	137	10	99.3
		40% VI		17.8	82	0.13	0.67
LNMF	(satin)	PET	continuous	∞	177	141	100
			*texturized*	15.5	87	0.19	0.4
MIKMF	mikado (twill)	PET	continuous	∞	290	165	100
			*texturized, weft parallel*	27.2	36	0.33	0.5
MOUMF	plaid double (twill)	70% acrylic	staple	30–38	1621	580	98.9
		30% WO	*carded WO*	18.6	1065	1.9	0.5
PAMF	flannel (twill)	45% PET	staple	35–38	788	275	100
		55% WO	*carded WO*	20.3	952	0.56	0.67
PEMF	percale (taffeta)	40% PET	staple	25	348	195	95
		60% CO	*open end yarn*	14.8	348	0.34	1
POPMF	poplin (taffeta)	80% PET	staple	45	135	112	84.6
		20% CO	*blended, carded CO*	11.7	135	0.18	1
TWEMF	tweed (satin)	60% PET	staple	32–38	4700	566	97.6
		20% CO		24.5	6000	1.2	0.5
		20% acrylic					
WFMF	plaid (twill)	60% PET	staple	25–38	803	280	99
		15% CO	*open end yarn*	19.2	803	0.85	0.67
		25% acrylic					

a Units: mm.

b Units: μm.

c Units: g 10^–4^ m^–1^ (dtex).

d Units: g m^–2^.

The compositional and dimensional features of fabrics, fibers, and yarns were partially obtained from the provider and completed with microscopical analysis using a digital lens, as well as measurements of the mass in an analytical balance. Table 1 includes some of these features. *Coverage factor* of the fabric is defined as the ratio of surface area actually covered by yarns to the total fabric surface area: the higher the coverage, the more compact the structure. *Binding coefficient* refers to the degree of interlacing of the yarns among the fabric, being 1 for plain fabrics, the most interlaced ones.

### 2.2 Fiber Release Experiments

Pilling experiments were performed in a random tumble pilling tester machine, based on the procedure of the ISO 12945-3:2020 Textiles–Determination of fabric propensity to surface pilling, fuzzing, or matting,^16^ a method recommended by the manufacturer. This machine aims to replicate the random wear that a textile will encounter during actual use. In the random tumble pilling tester, stainless-steel rotors tumble unmounted fabric samples within a cork-lined chamber assisted by compressed air. This allows subjecting the textiles to a wearing program according to the aforementioned standard. While based on that procedure, the intervals of testing were adapted to our sampling needs. The global time of testing, 60 min, was divided in 7 steps: 1, 3, 3, 3, 10, 20, and 20 min. Samples were weighed at the end of each of these steps. Figure 2 represents the evolution of the experiment regarding the tested samples. Initially, the specimens labeled as 1, 2, and 3 are introduced in the drum. After 4 min of testing, specimen 1 is substituted by specimen 4, and in the minute 10, specimen 2 is substituted by specimen 5. This method is based on the alternative procedure contemplated in ISO 12945-3:2020 in order to optimize the testing time.

**Figure 2:**
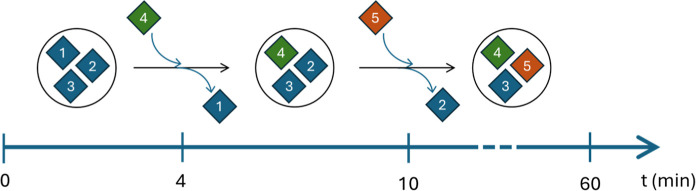
Diagram of the content of the drum during the fiber release experiments, pointing the change of specimen 1 by specimen 4 after 4 min testing, and the change of specimen 2 by specimen 5 after 10 min testing.

#### 2.2.1 Sample Preparation

Squared specimens of 10 × 10 cm^2^ were cut in an angle of 45° with respect to the warp and the weft, and the edges were glued and let dry for 24 h. The room air was conditioned for at least 1 h before the experiments. The samples were weighed just after being removed from the machine to check for minimum moisture absorption.

### 2.3 Measurement of Dynamic and Thermo-Mechanical Properties

Strain and frequency sweeps and temperature ramps were performed using a tensile geometry in a Rheometrics dynamic mechanical thermal analyzer (DMTA)-IV instrument, using rectangular pieces of the fabrics of 5 mm length and 4 to 6.5 mm width. Figure 3A represents the clamps used in the tensile tests. Both strain and frequency sweeps consist of applying a sinusoidal deformation to the samples, thus measuring the stress. In strain sweep, the amplitude of the deformation is increased in time, while in the frequency sweep, the amplitude remains constant but the frequency of deformation decreases. Figure 3B,C represents the evolution of the strain with time in both frequency and strain sweeps. Frequency sweeps, performed at room *T* (around 25 °C), consisted of ramps of 100–0.1 Hz at 0.01% strain with 10 N of initial static force. Temperature ramps were performed at 3 °C/min, starting at room *T* (around 30 °C) up to 60 °C. These ramps were performed in a transient mode, applying a constant stress of 10 kPa. All mechanical tests were repeated with tension applied in both the warp and weft directions.

**Figure 3:**
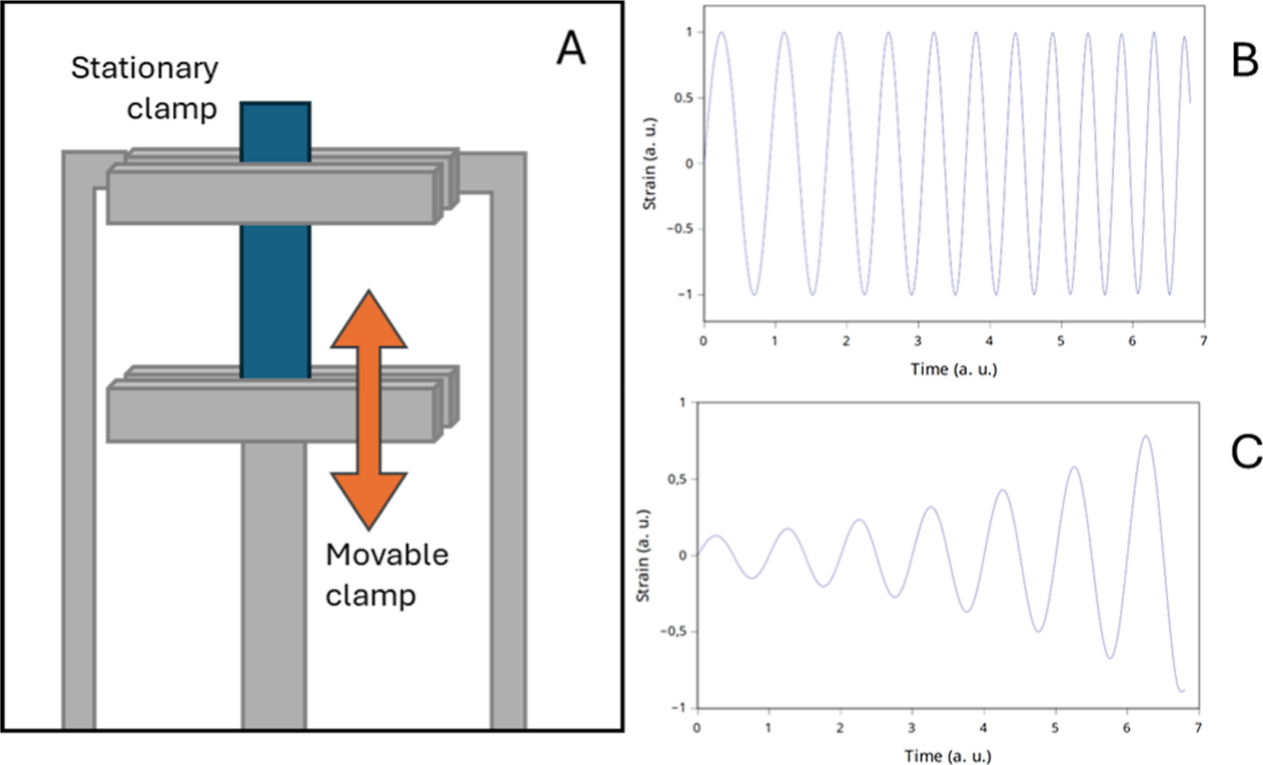
(A) Schematic representation of the tensile geometry in DMTA-IV tests; and graphical representation of the strain evolution in arbitrary units with time in (B) frequency sweep and in (C) strain sweep.

### 2.4 Analysis of the Data

The processing of the data and the generation of the graphics for the statistical analyses was performed in software RStudio.^17^ The variables analyzed in this study were compared using PCA, through the function PCA­() from the R package “FactoMineR”. The visualization of the results was made by using the fviz_pca­() functions from the R package “Factoextra”. The evaluation of statistically significant differences in fiber release was performed through one-way ANOVA test with 5% significance level, using the function aov­() from the package “stats”, and the boxplot was performed using the function ggplot­() from the R package “ggplot2”. Further information about the R functions used in the present study can be found in the Supporting Information.

## 3 Results and Discussion

### 3.1 Pilling Experiments

The mass loss after each step of a pilling experiment was measured by weighing the samples and calculating the mass difference compared to the value at the very beginning of the experiment. An ANOVA test was performed to determine if there were statistically significant differences between the final release of the fabrics after 60 min of testing. The results are presented in Table 2, where Df means degrees of freedom, sum sq is the sum of the squares, and mean sq is the mean of the squares. The mean square of the release divided by the same value for the residuals is the observed test statistic for the *F* test, the *F* value. The observed *F*-value is higher than that tabulated one for 15 variables and 3 replicates, which is 2.03742. That indicates a higher variance between the fabrics than that within the replicates, revealing that all fabrics present statistically significant release differences. The *p*-value, which is lower than 0.05, agrees with this statement.

**Table 2:** ANOVA Results in R Function aov­()

	Df	sum sq	mean sq	*F* value	*p*-value
release	14	76,681	5477	241	<2 × 10^–16^
resuduals	30	682	23		

Figure 4 shows a boxplot diagram of the fiber release measured for the three replicates of each fabric performed in those fiber release experiments, comparing the release after 60 min testing. The fabric with the higher release is POPMF, followed by MOUMF, TWEMF, and WFMF. The rest of the fabrics present less than 50 mg of fibers lost per gram of fabric.

**Figure 4:**
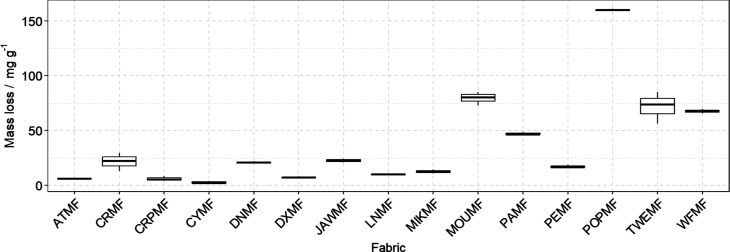
Boxplot diagram for the three replicates of the fiber release of the 15 fabrics after 60 min testing.

It is noticeable that the fabrics exhibiting the higher mass losses are all composed by a natural fiber mixed with PET and/or acrylic, and that the ones producing the least amount of released fibers are made of PET and EL (CYMF, ATMF, CRPMF), or 100% PET. Dos Santos et al.^18^ studied the influence of the composition in the abrasion resistance of the fabrics, concluding that the main variable in this behavior is the composition when studying PET and CO fibers. However, it should be mentioned that although PET fibers are produced as continuous filaments and used in this way when they are used alone, they are usually cut when mixed with staple fibers such as CO.

When representing the fiber loss in front of time, using the measurements made after each step of the tests, a power trend has been found, following eq 1.

1 $$\Delta m=at^{b}$$

Eq 1 shows the power model for the fiber loss behavior of the fabrics in the pilling experiments.

Δ*m* is the mass loss divided by the initial mass, measured in mg/g, *t* is the testing time in min, *a* is the parameter that define the rate of initial loss, and *b* is the capacity to stabilize the mass after long testing time. The higher the *b* values, the higher the capacity to stabilize the mass loss at long testing times.

The experiments were performed in triplicate, placing one sample consisting of three specimens in each drum. Figure 5 represents the data obtained for the mass loss of the fabric DNMF, with one series per drum. The power trend, represented as a black line, was calculated using the average values of the three series.

**Figure 5:**
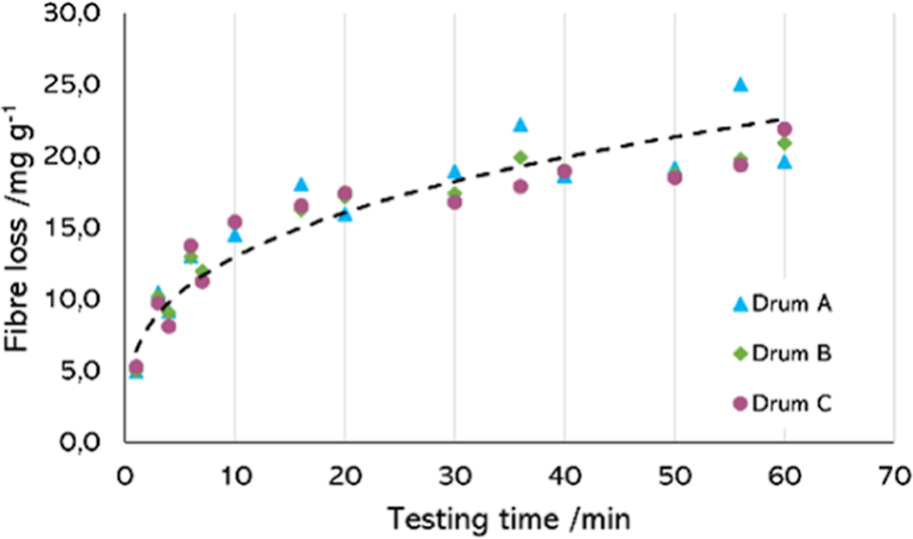
Fiber release of the three replicates of the DNMF fabrics performed in the random tumble pilling tester, with the power trend of the data.

Figure 6 shows an overlay of the power trends calculated for all fabrics. In all cases, a sharp initial loss can be seen in the first 5–10 min. Then, the release tends to stabilize, reaching an almost constant value. The initial mass loss rate is defined by the component *a* of eq 1, being higher for the fabrics POPMF, MOUMF, WFMF, and TWEMF, which are fabrics made of staple fibers, all containing at least 20% of a natural fiber blended with PET and/or acrylic. On the other hand, the lowest values of *a* correspond to CYMF, CRPMF, ATMF, and LNMF. These fabrics are made of continuous, artificial fibers. In LNMF, PET is found alone, while in the others, it is blended with EL and, in the case of CYMF, also with acrylic.

**Figure 6:**
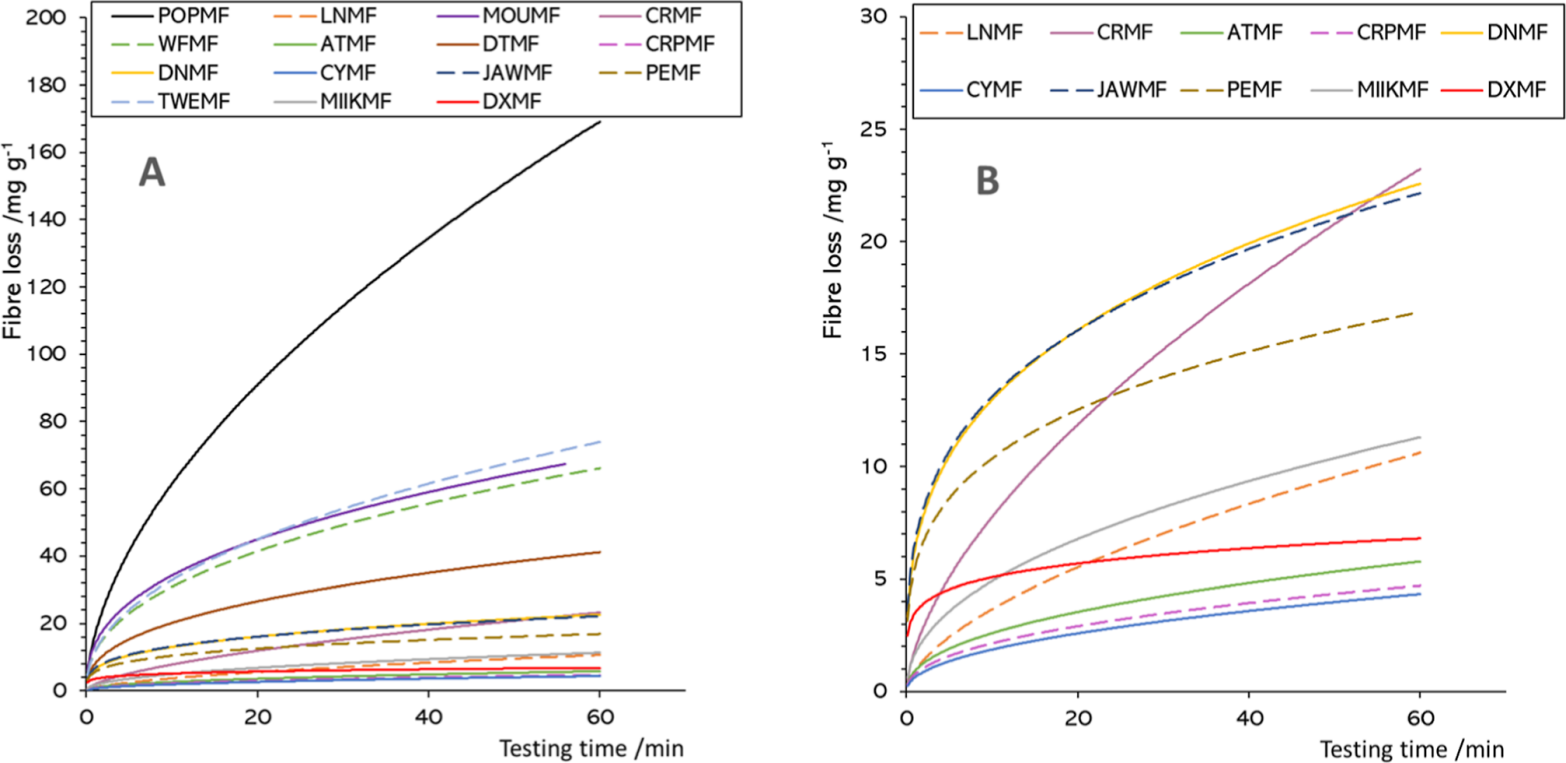
Overlay of the power models based on the pilling experiments results for (A) all fabrics and (B) only the fabrics that showed the lowest release.

When considering the trend at longer times, above 15–20 min, most fabrics present similar slopes, or values of the coefficient *b*. DXMF, PEMF, JAWMF, and DNMF are the fabrics with the lowest value of *b*. In this case, JAWMF is made of continuous fibers, while the others are staple. However, all are composed of artificial fibers. The fabrics with the highest values of *b*, in other words, those which stop releasing fibers at longer testing time, are CRMF, LNMF, and POPMF. The two formers are made of 100% continuous PET fibers, while POPMF contains staple PET fibers blended with 20% of CO.

This difference between continuous and staple filaments has been studied in similar pilling tests by Dalla Fontana et al.,^10^ who found that textiles with continuous PET filaments release more fibers than those made of staple fibers. Comparing with the current study, that behavior seems to be related to the *b* parameter of our model. This is compatible with their explanation of staple fibers forming pills that remain attached to the fabrics, while continuous fibers have less tendency to form pills.

The data referred to the power model fitting are displayed in Table 3, including the parameters *a* and *b*, and the squared residuals of the models. Figure S1 in the Supporting Information shows the power model of each fabric together with the average of the mass loss obtained in the three drums. As can be seen, all samples except CYMF and DXMF showed a good fit, with *R* ^2^ values close to 1. The lower fit considering the data could be due to moisture sorption from the environment during the weighing process. These fabrics could absorb moisture faster than the others due to different factors such as composition, weave pattern, or other fabric characteristics making that these specific fabrics do not follow the fitting model. In the case of CYMF, as it is a corduroy, it presents a higher available surface. Further studies could compare the moisture sorption rate of these fabrics.

**Table 3:** Parameters and Error of the Exponential Model for the Pilling Experiments

	ATMF	CRMF	CRPMF	CYMF	DNMF	DTMF	JAWMF	LNMF
*A*	0,9322	1,9035	0,7833	0,6360	6,3664	8,0438	6,6850	0,9352
*B*	0,4457	0,6111	0,4380	0,4689	0,3092	0,3986	0,2927	0,5935
R^^2^	0,9674	0,9805	0,9808	0,7897	0,9207	0,9564	0,9728	0,9737

	MIKMF	MOUMF	PEMF	POPMF	TWEMF	WFMF	DXMF
*A*	1,6909	13 824	5,5667	16 703	11 608	11 555	3306
*B*	0,4641	0,3935	0,2708	0,5655	0,4522	0,4261	0,2045
R^^2^	0,9759	0,9879	0,9537	0,9815	0,9598	0,9942	0,7494

As it is shown, the error of the model is negligible except for two fabrics, which have the lowest fiber losses, CYMF and DXMF.

### 3.2 Dynamic and Thermo-Mechanical Properties

#### 3.2.1 Frequency Sweeps

The value of the storage modulus of the fabrics was measured through a range of frequencies. The fabrics were tested in both the warp and weft directions. Figure 7 shows typical plots of the storage modulus vs frequency in the warp and weft directions. It can be seen how even the two yarns are supposed to be the same; their storage moduli present different behaviors, probably because of the loads at which they are subjected during manufacture.

**Figure 7:**
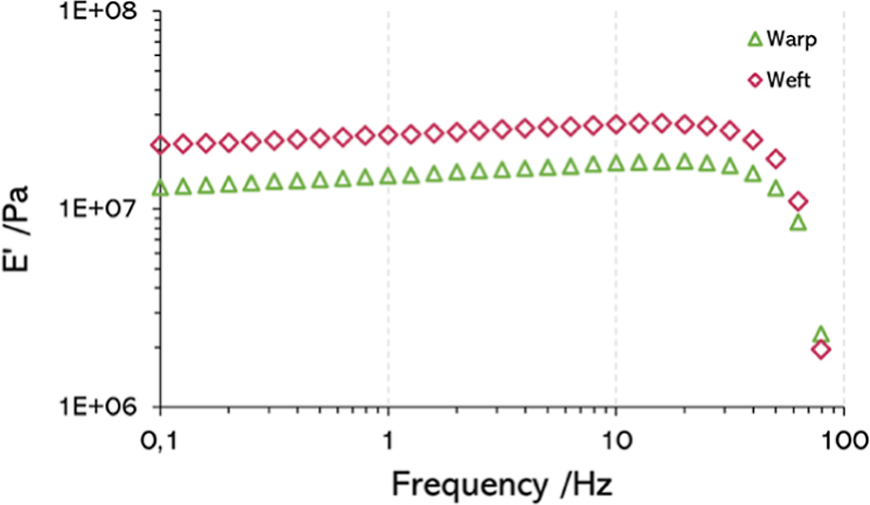
Storage modulus evolution in a frequency sweep of the MOUMF reverse fabric, tested in warp and weft directions.

The results of our measurements are represented in Figure 9, together with the average value. MOUMF, WFMF, and PEMF, Figure 8, have a pattern made of different stripes with yarns of different colors. Thus, each stripe was tested separately, and a weighted average of the results was calculated for the entire fabric. The weighted average considers the width of each stripe present in the fabric.

**Figure 8:**
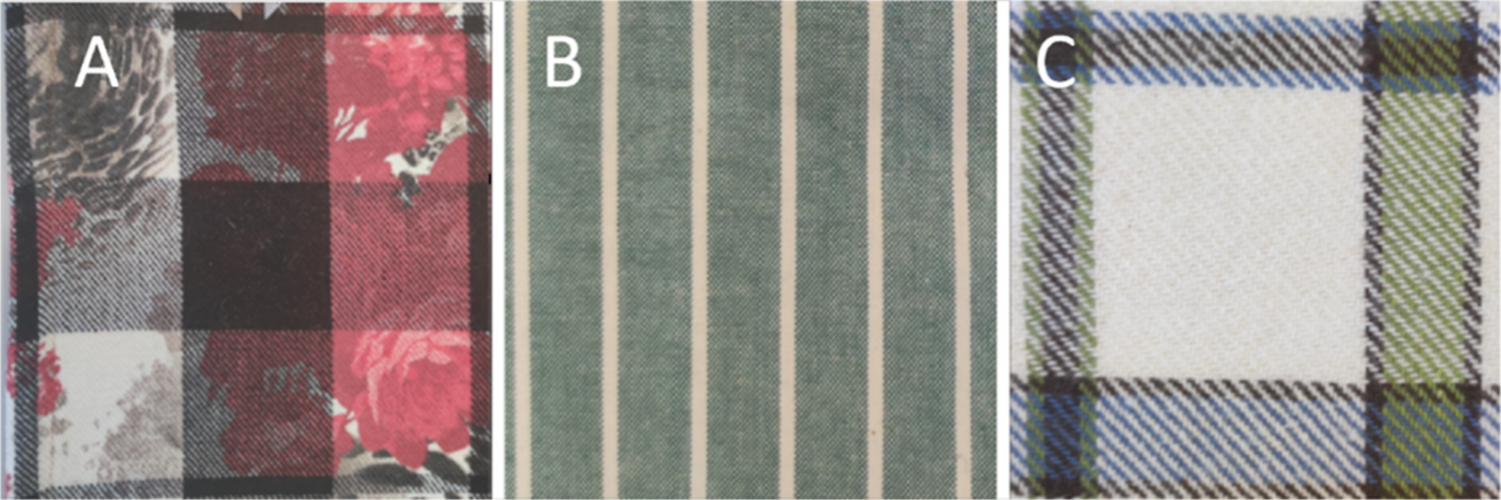
Images of the (A) MOUMF, (B) PEMF, and (C) WFMF showing their stripe patterns made of different yarns.

Figure 9 shows the differences between fabrics and also within them, considering both directions of the yarns, warp and weft. It is interesting to notice that even in fabrics manufactured with the same yarns in warp and weft, like POPMF, DXMF, or PAMF, it is possible to find differences in the behavior considering the testing direction. This could arise from the mechanical loads at which yarns were subjected during manufacture, which were different for warp and weft. Other fabrics with the same yarns in warp and weft, such as CRPMF do not present that variation.

**Figure 9:**
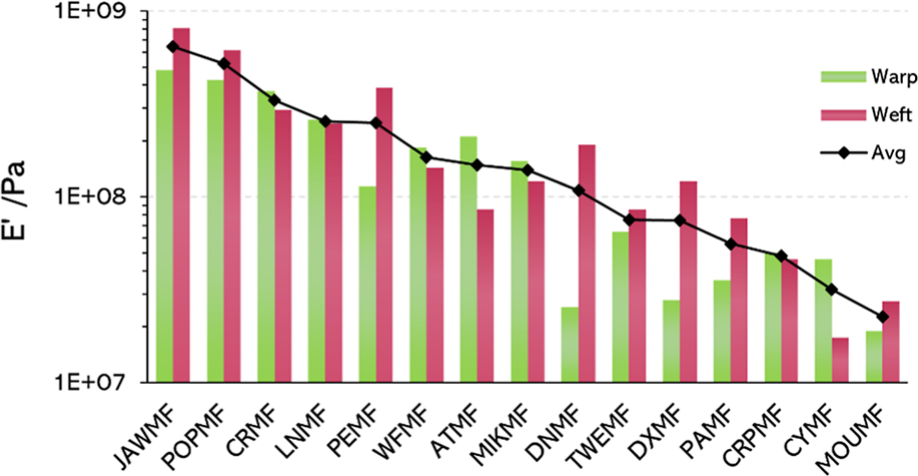
Storage modulus of the fabrics measured through frequency sweep.

When analyzing the global value of the storage modulus, the black line in Figure 9, it is noticeable that the four most rigid fabrics present high content in PET: three of them are 100% PET, while the other is 80% PET, blended with CO. On the other side, the more elastic ones have different compositions. MOUMF and PAMF contain WO, which has a natural crimp, as it is a protein with α-helix structure. CYMF and CRPMF present EL, which is an elastomer, and therefore, as expected, their contribution to *E*′ appears small.

#### 3.2.2 Temperature Ramps

The fabrics were subjected to temperature ramps from room conditions up to 60 °C, while a constant tensile stress was applied and the strain was measured. PEMF and TWEMF stripes were tested separately, and MOUMF was tested by cutting stripes half-black-half-white. Additionally, as the MOUMF consists of two fabrics sewed together, both layers were separated and tested separately one from the other. Figure 10 represents the elongation behavior for two fabrics in both warp and weft directions. The plots of the other fabrics can be seen in Figure S2 of the Supporting Information. The fabrics tend to elongate as the temperature rises. Most of them follow an almost linear tendency after the first moments of the heating. The order of the elongation is similar for all of them, except for MOUMF and JAWMF which are deformed more than 1% (notice that the scales in those cases are different to appreciate the slopes).

**Figure 10:**
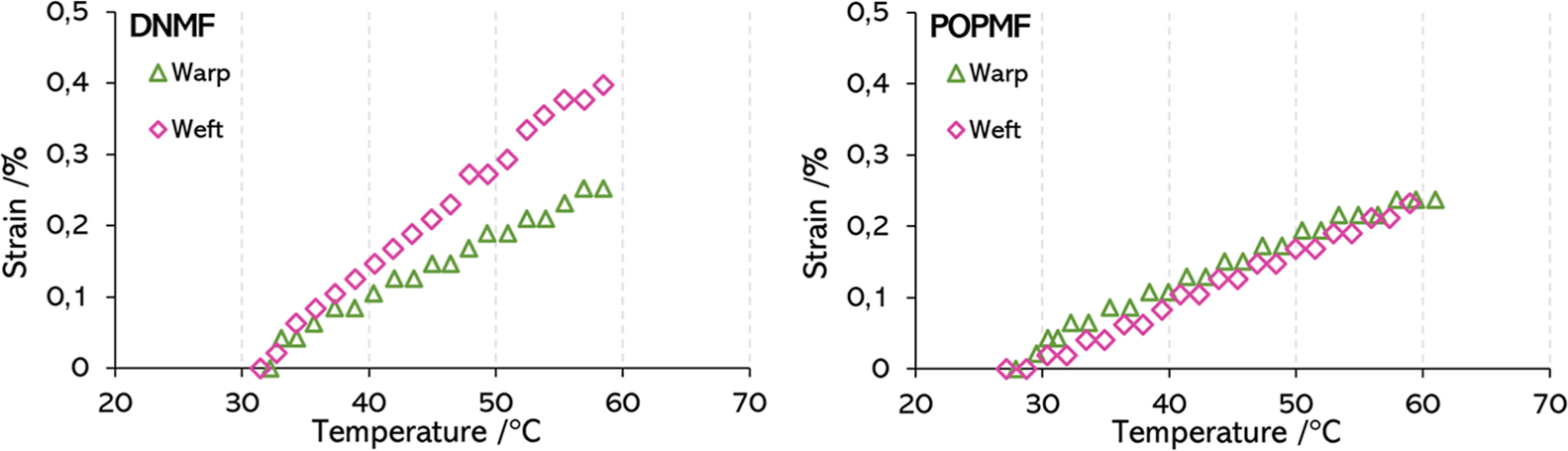
Evolution of the strain in front of temperature for the fabrics DNMF and POPMF. The warp is represented in green and the weft in pink.

The warp and weft yarns of DNMF are different, while in POPMF, all yarns are the same. As it is supposed, the yarns of DNMF present different elongation rates. In the case of POPMF, the signals in both directions are more alike; however, they are not exactly overlapped. The slightly lower elongation of the weft yarns can be a result of the differences in the mechanical loads applied in each direction during manufacture. In general, for fabrics with the same yarns in both warp and weft, the elongation is higher for the warp. In some cases, such as PAMF, LNMF, CRPMF, and POPMF, the difference arises from the lower temperatures, where the weft begins to elongate at higher temperature and then follows approximately the same deformation rate as the warp. On the other hand, the higher deformation of the warp in other fabrics arises from the rate of deformation, higher in the warp than that in the weft direction. This is the case of CRMF, the undyed yarns of PEMF and WFMF, and the yarns in the face layer of MOUMF. The exceptions of this behavior are CYMF, the reverse layer of MOUMF, and the dyed yarns of WFMF, which show a higher deformation in the weft yarns.

### 3.3 Statistical Analysis. Principal Component Analysis

The release of fibers in the fabrics can be influenced by so many variables that a proper data processing becomes essential. Considering each variable as a different dimension, the optimal way to visualize them all is to perform a PCA, projecting the variables in the orthogonal directions of maximum variance.

#### 3.3.1 Variables and Individuals

The analysis studies 14 features (variables) of the 15 fabrics (individuals). Among the selected variables, there are features associated with the nature of the fibers, either natural or artificial. Other group of variables related to physical dimensions, such as fiber length and its diameter, the yarn linear density, known as yarn count, the fabric area density or grammage, the fabric thickness, the coverage factor of such textile, and its binding coefficient. The thermo-mechanical properties of the textiles are represented by the storage modulus measured in frequency sweeps and by the strain produced at 50 °C in the temperature ramps. Finally, the variables related to fiber release test results are the *a* and *b* coefficients of the dry release equation models, corresponding to the number of fibers released at the beginning of the test, the *initial release*, and the ability to continue releasing fibers at long testing times, the *release at long time*. Additionally, the materials were supplied with wet release data obtained in a 30 min wash at 40 °C in a laboratory washing machine.

#### 3.3.2 Principal Component Analysis Results

In the first step of the analysis, the orthogonal directions of maximum variance in the space, known as the principal components, are calculated. Figure 11 represents the percentage of explained variance of the principal components. These percentages are the eigenvalues of the directions. The two main components can only explain the 53.7% of the variance, which is not enough to discard all the others. Therefore, the third and fourth components must be analyzed as well in order to ensure that almost 80% of the variance is considered. However, as it is impossible to plot in four dimensions, they will be represented separately.

**Figure 11:**
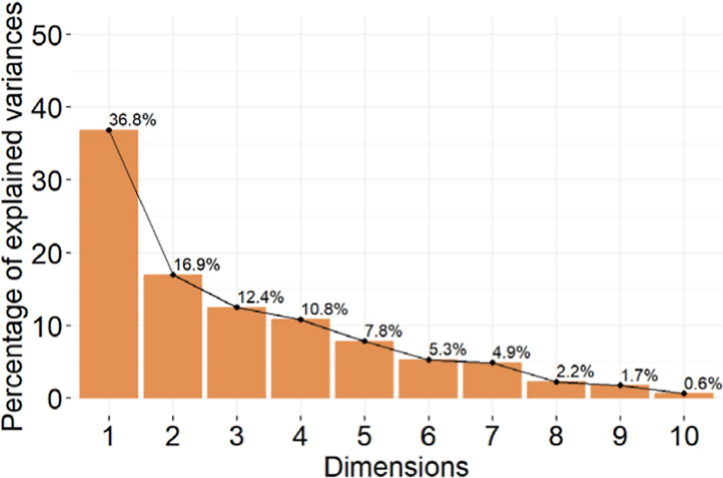
Percentage of explained variance of the principal components.

Figure 12 shows the contributions of the PCA variables to the principal components. The most important variables in PC1 and PC2 are the ones related to the density of the fabric, grammage and thickness, the fiber length and the nature of the fibers, as well as the initial release in dry conditions and the wet release. In the third and fourth dimensions, the mechanical properties become more important, together with the release at long time, the coverage factor, and the fiber diameter.

**Figure 12:**
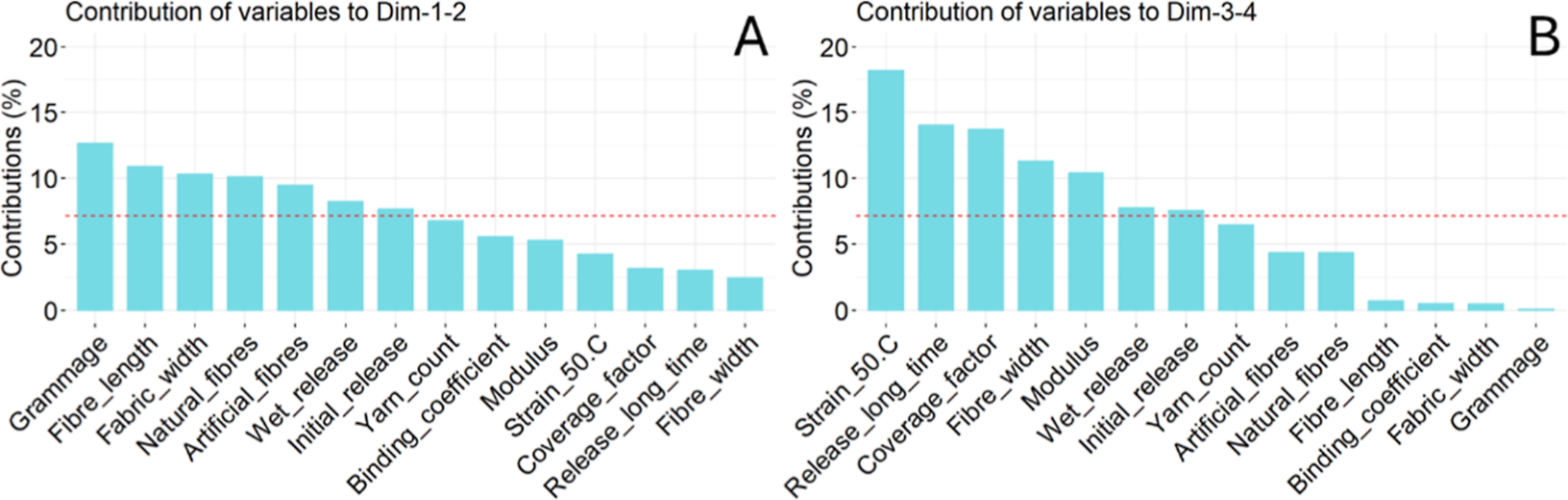
Contributions of the PCA variables to (A) dimensions 1 and 2, and (B) dimensions 3 and 4.

Knowing the weight of the variables in the PCs, in order to understand the relationship between them, a PCA plot must be analyzed. Figure 13 shows the PCA variables represented in the principal component directions, PC1 and PC2, with the arrows colored according to the contribution to PC1. In this plot, the variables whose arrows are represented in similar or opposite directions are the most related, while the perpendicular arrows correspond to variables that are independent of each other. Moreover, the longer the arrows, the higher their contribution to the principal components.

**Figure 13:**
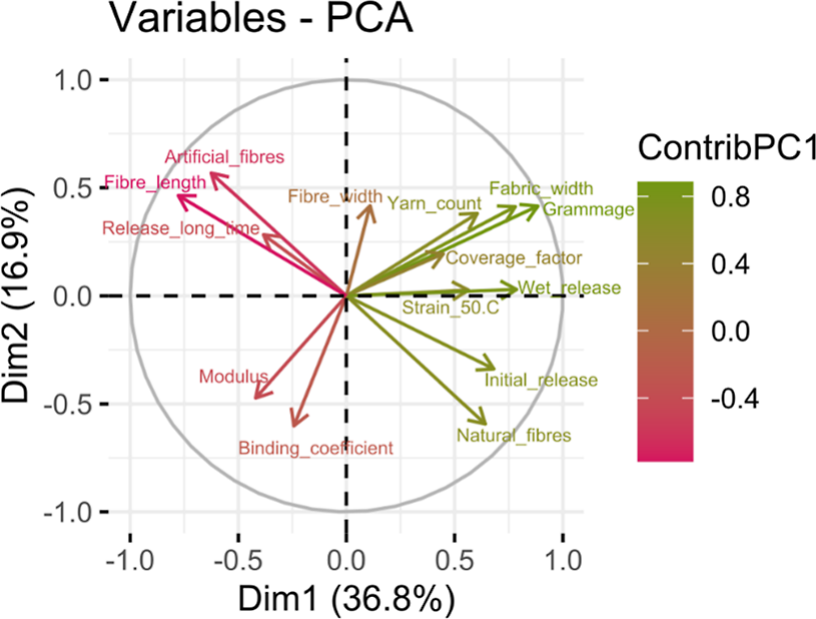
PCA variables represented in the principal component directions, with the arrows colored according to their contribution to PC1.

Considering the two coefficients of the dry release model, the initial release and the release at long time, it is noticeable that they are almost aligned in opposite directions, which suggests that those fabrics that shed the most under the first abrasion become more stable at a long testing time. Considering the most related variables with this dry release, natural fibers are related to an initial release while the artificial ones are related with the release at long testing time. In relation to the fiber length, it is worth mentioning that the longer the fiber, the lower the initial release.

An interesting relation is observed between the wet release data and the DMA constant stress ramp temperature results. The vector of the strain obtained at 50 °C follows almost the same direction as the wet release arrow on the PCA plot. This observation points to a possible relationship between the fabric elongation capacity of the fabric and shedding during washing at a similar temperature.

After the most important directions of variance were considered, the two principal components are studied. As shown before in Figure 12, some of the variables in PC3 and PC4 present small contribution, becoming nearly negligible for the analysis of the plot. Thus, in Figure 14, only the main PCA variables in the dimensions PC3 and PC4 are represented.

**Figure 14:**
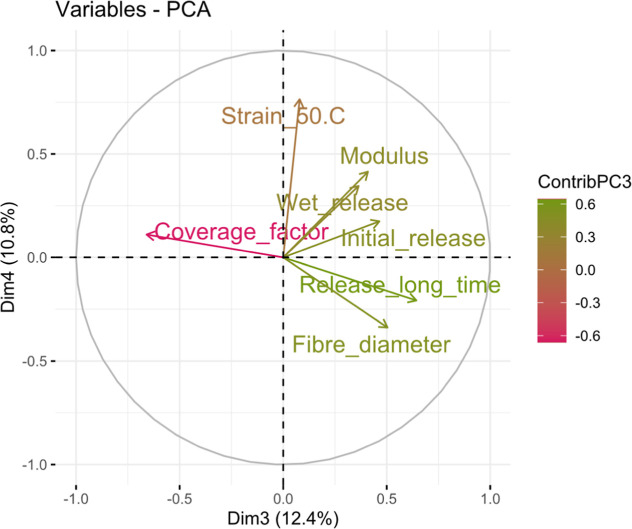
Variables with greater contribution represented in PC3 and PC4, with the arrows colored according to their contribution to PC1.

In these directions, both wet release and initial dry release are strongly related to the fabric modulus, pointing to a contribution of the fabric stiffness to the shedding effect. In this case, the release at long time appears to be more related with the fiber diameter and the coverage factor of the fabrics. Thus, those textiles with a large percentage of uncovered area and coarse fibers would continue to release fibers at higher rates over long periods of time.

## 4 Conclusions

This study evaluated different features regarding textiles in order to determine whether they are related with microfiber emissions during pilling experiments and washing. The fiber release experiments, which aim to simulate the wear and tear of the garments over a long time, present a power trend pointing to a higher release of the fibers in the first steps, followed by a relatively stabilization of the mass at longer testing times.

The principal components of PCA analysis show that dry release is affected mostly by the nature and length of the fibers, as an initial loss is promoted in short, natural fibers, while on the other hand those fabrics made of longer, artificial fibers tend to continue shedding at long-term. Wet release presents more dependence on thermo-mechanical properties and yarn structure as it is more related to the storage modulus and the strain at 50 °C under stress, together with the yarn count, the fabric thickness, and grammage of the fabric.

## Supplementary Material



## Notes

R functions: brief description of R functions, analysis of the data, ANOVA test, visualization of boxplot, PCA, visualization of PCA results, fiber release data: average of the mass loss obtained in the three drums with the power model of each fabric, and temperature ramp results in DMTA: evolution of strain in front of temperature (PDF)
