# Supplementary material for: Textile Fiber Pollution: Relating Textile Features to Fiber Release in Pilling Experiments
Source: ACS Omega. 2025 May 29;10(22):22472–81. doi: 10.1021/acsomega.4c09501 (PMC12163674; doi:10.1021/acsomega.4c09501)
Supplement: Supplementary file 1 [file ao4c09501_si_001.pdf]

TEXTILE FIBER POLLUTION: RELATING TEXTILE FEATURES TO FIBER RELEASE IN PILLING EXPERIMENTS

Mercedes Pereira[1], Jorge López-Beceiro[1*], Ana-María Díaz-Díaz[1], Laura S. Vázquez[1], Ramón Artiaga[1]

[1] Centro de Investigacións en Tecnoloxías Navais e Industriais, Universidade da Coruña, Campus Industrial de Ferrol, Ferrol, 15403, Spain
* E-mail: jorge.lopez.beceiro@udc.es

R Functions

The R functions used in this study were applied to the statistical analysis of the data. A brief explanation of each of them is included in the present appendix:

ANOVA test
Function aov()
Package “stats”

The fitting of an Analysis of variance model, by setting a formula to specify the model, applied to a data frame. In this case, the data are replicates of the fiber release measurements. The test compares the variance between fabric replicates and the variance between fabrics.

Chambers, J. M., Freeny, A and Heiberger, R. M. (1992) Analysis of variance; designed experiments. Chapter 5 of Statistical Models in S eds J. M. Chambers and T. J. Hastie, Wadsworth & Brooks/Cole

Visualization of Boxplot
Function ggplot()
Package “ggplot2”
“ggplot() initializes a ggplot object. It can be used to declare the input data frame for a graphic and to specify the set of plot aesthetics intended to be common throughout all subsequent layers unless specifically overridden.”

Principal Component Analysis
Function PCA()
Package “FactoMineR”

“Performs Principal Component Analysis (PCA) with supplementary individuals, supplementary quantitative variables and supplementary categorical variables. Missing values are replaced by the column mean.”
Husson, F., Le, S. and Pages, J. (2010). Exploratory Multivariate Analysis by Example Using R, Chapman and Hall.

Visualization of PCA results
Function: fviz_pca()
Package “Factoextra”

fviz_pca() provides ggplot2-based visualization of PCA outputs from PCA (in FactoMineR), among other PCA functions.

Fiber release data

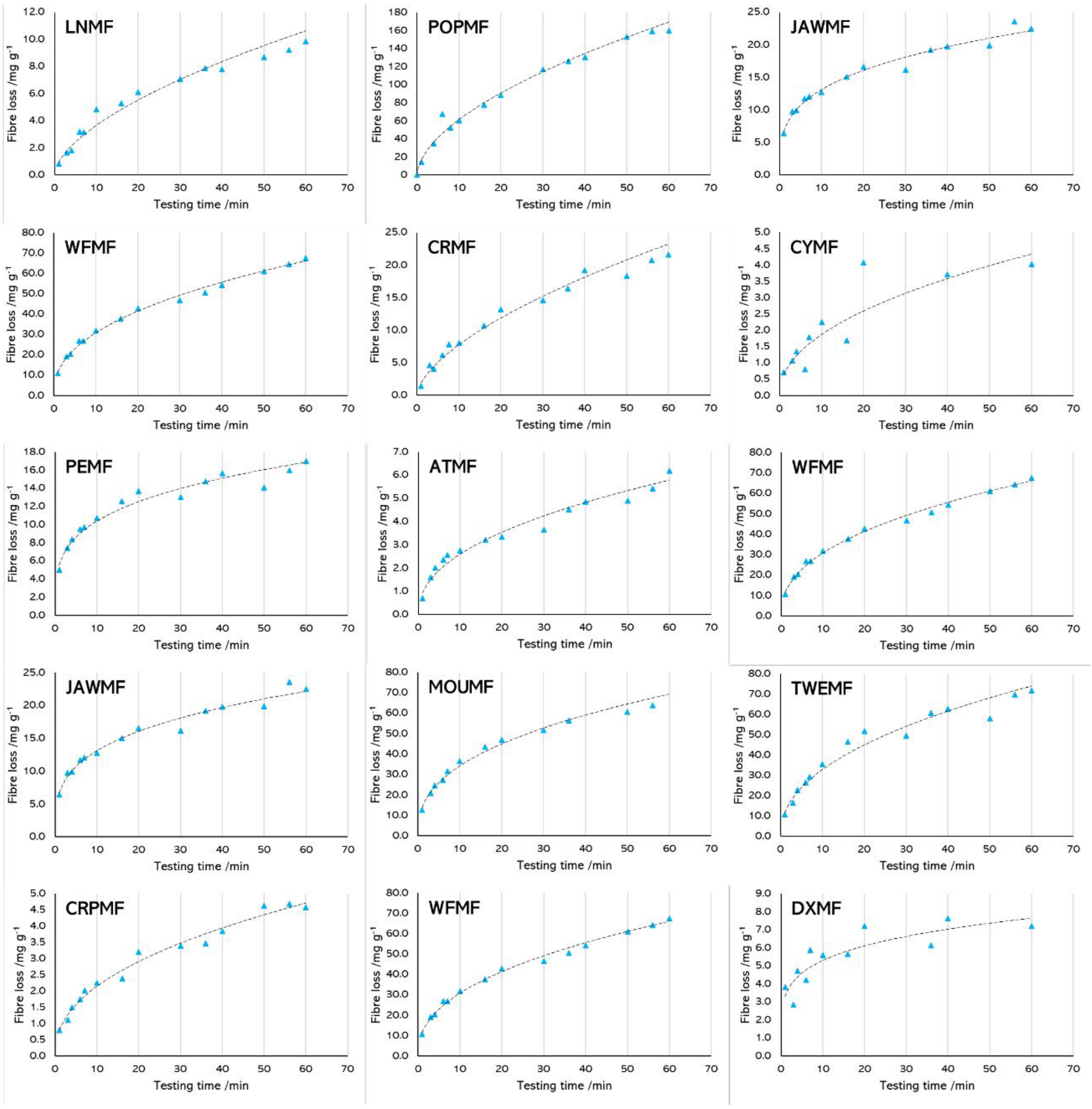

Figure S1 Evolution of the accumulated mass loss of each fabric, considering the average of the samples in the three drums, together with the power model for each fabric.

Temperature ramp results in DMTA

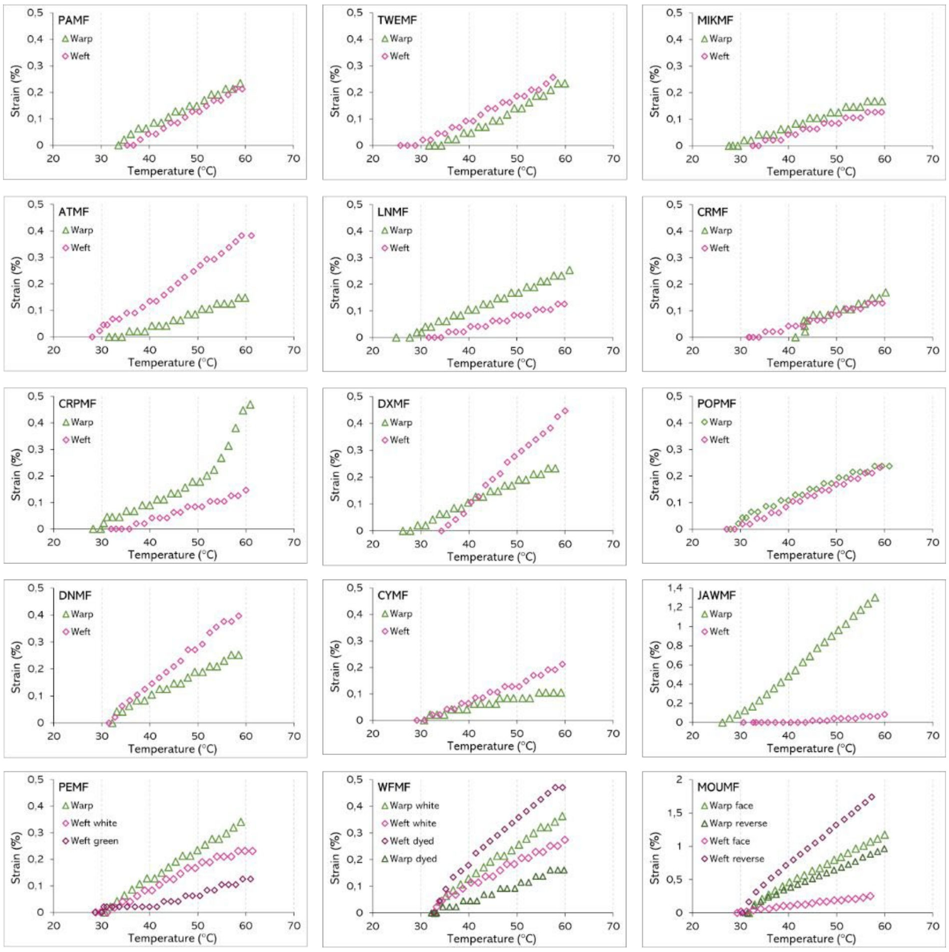

Figure S2 Evolution of the strain in front of temperature for the fabrics, with the warp in green and weft in pink.
